# Pig Movement Estimation by Integrating Optical Flow with a Multi-Object Tracking Model

**DOI:** 10.3390/s23239499

**Published:** 2023-11-29

**Authors:** Heng Zhou, Seyeon Chung, Junaid Khan Kakar, Sang Cheol Kim, Hyongsuk Kim

**Affiliations:** 1 Department of Electronics and Information Engineering, Jeonbuk National University, Jeonju 54896, Republic of Korea; hengz@jbnu.ac.kr (H.Z.); junaidk@jbnu.ac.kr (J.K.K.); 2 Core Research Institute of Intelligent Robots, Jeonbuk National University, Jeonju 54896, Republic of Korea; nuguriie@gmail.com (S.C.); sckim7777@gmail.com (S.C.K.); 3 Department of Electronics Engineering, Jeonbuk National University, Jeonju 54896, Republic of Korea

**Keywords:** pig movement estimation, multi-object tracking, optical flow, livestock farming

## Abstract

Pig husbandry constitutes a significant segment within the broader framework of livestock farming, with porcine well-being emerging as a paramount concern due to its direct implications on pig breeding and production. An easily observable proxy for assessing the health of pigs lies in their daily patterns of movement. The daily movement patterns of pigs can be used as an indicator of their health, in which more active pigs are usually healthier than those who are not active, providing farmers with knowledge of identifying pigs’ health state before they become sick or their condition becomes life-threatening. However, the conventional means of estimating pig mobility largely rely on manual observations by farmers, which is impractical in the context of contemporary centralized and extensive pig farming operations. In response to these challenges, multi-object tracking and pig behavior methods are adopted to monitor pig health and welfare closely. Regrettably, these existing methods frequently fall short of providing precise and quantified measurements of movement distance, thereby yielding a rudimentary metric for assessing pig health. This paper proposes a novel approach that integrates optical flow and a multi-object tracking algorithm to more accurately gauge pig movement based on both qualitative and quantitative analyses of the shortcomings of solely relying on tracking algorithms. The optical flow records accurate movement between two consecutive frames and the multi-object tracking algorithm offers individual tracks for each pig. By combining optical flow and the tracking algorithm, our approach can accurately estimate each pig’s movement. Moreover, the incorporation of optical flow affords the capacity to discern partial movements, such as instances where only the pig’s head is in motion while the remainder of its body remains stationary. The experimental results show that the proposed method has superiority over the method of solely using tracking results, i.e., bounding boxes. The reason is that the movement calculated based on bounding boxes is easily affected by the size fluctuation while the optical flow data can avoid these drawbacks and even provide more fine-grained motion information. The virtues inherent in the proposed method culminate in the provision of more accurate and comprehensive information, thus enhancing the efficacy of decision-making and management processes within the realm of pig farming.

## 1. Introduction

The increasing integration of Artificial Intelligence (AI) into the agricultural sector has garnered significant attention recently, primarily propelled by the rapid advancements in AI technologies. Within the domain of computer vision, recognized for its intricate tasks spanning object detection, action recognition, multi-object tracking, and more, AI has demonstrated successful applications across diverse agricultural domains. These applications encompass crucial areas such as plant disease detection [1], pig behavior recognition [2], cattle behavior recognition [3], and livestock tracking [4], among others. It is paramount to underscore the pivotal role of livestock farming in the broader agricultural landscape, serving as a primary source of meat production for a significant portion of the global population. In response to this evolving agricultural paradigm, a novel concept known as Precision Livestock Farming (PLF) has emerged. PLF harnesses the synergistic capabilities of AI and Internet of Things (IoT) technologies, equipping livestock farmers with scientifically informed decision-making tools and adaptive management strategies [5,6]. This innovative approach ushers in a new era in livestock farming, where data-driven insights and intelligent systems empower farmers to optimize their operations and enhance the overall efficiency and sustainability of livestock production.

Pig health stands as a recurrent focal point within the realm of livestock farming given its intricate interplay with pig breeding and production. A fundamental yardstick for assessing pig well-being lies in the daily ambulatory patterns exhibited by pigs. In conventional pig farming, the onus of monitoring pig movement typically falls upon farmers, an endeavor demanding substantial time and labor resources [7,8]. However, modern commercial pig breeding enterprises have embraced a centralized and large-scale operational paradigm, rendering traditional labor-intensive monitoring approaches impractical. Moreover, the demanding working conditions pervasive in the pig farming industry have restricted the pool of individuals willing to pursue careers in this sector, resulting in a dearth of labor resources for monitoring pig movement. These factors underscore the pressing necessity for automated approaches to pig farming.

In response, numerous researchers have dedicated their efforts to this burgeoning field of study. Broadly, two predominant methodologies for estimating pig movement have emerged. The first approach leverages behavior recognition algorithms [9,10,11,12] to classify various pig activities, encompassing lying, walking, sitting, standing, drinking, etc. The second approach harnesses tracking algorithms [8,13,14] within the domain of computer vision to monitor the positions of individual pigs and subsequently assess pig movement using the center point of bounding boxes. Notably, two pivotal considerations underlie the process of calculating pig movement. The first key point is that the effectiveness of the tracking method is of paramount importance, necessitating a tracking model capable of consistent tracking across all frames for each pig. The second key point is how to obtain pig movement. Although significant strides have been made in the realm of pig movement assessment, existing methodologies exhibit limitations in providing both individual and cumulative distance measurements. Regarding the methods based on behavior recognition, this strand of research primarily concentrates on static behaviors and fails to provide quantified assessments of the current and cumulative movements of individual pigs, i.e., how far each pig moves and how long each pig keeps moving in one hour or one day. As for tracking-based approaches, the related literature tends to first track the pigs, obtaining bounding boxes for each pig, and then use the distance the center points of bounding boxes move as the measure of pig movement. Nevertheless, the accuracy of motion distance measurements in this method hinges on the size of bounding boxes, rendering calculations inaccurate when bounding box dimensions fluctuate due to tracking limitations.

**Figure 1:**
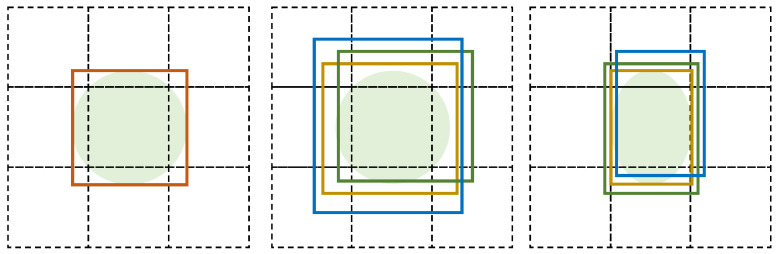
Illustration of the problem that bounding boxes are easily changed during tracking. The left sub-figure shows a bounding box in the current frame. The middle sub-figure indicates the bounding box in the next frame where all three kinds of bounding boxes might be the tracks of the object. The right sub-figure means a case that the object does not move, while its shape changes, resulting in the change in tracks.

This problem can be illustrated in Figure 1. In this illustration, the left sub-figure visually conveys a bounding box encapsulating a stationary object within the current frame. Meanwhile, the middle sub-figure illustrates three distinct bounding box scenarios for the same object in the subsequent frame. Because Intersection over Union (IoU) matching is the main measure in the tracking algorithm to associate the two bounding boxes of the same object in consecutive frames, bounding boxes characterized by high IoU values all bear a substantial likelihood of being considered valid tracking results. In this situation, all of those three bounding boxes could be the tracking results, while the coordinates of the center points of those three bounding boxes changed, even though the object is static, resulting in errors for estimation pig movement. Furthermore, the right sub-figure of Figure 1 presents another case where the object is static while its shape changes, which is very common for pigs. In this case, the sizes of the generated boding boxes are also changed. Consequently, it is not reliable to take the distance moved by the center point of the bounding box as the measurement of pig movement.

In light of the prevailing limitations inherent in extant research pertaining to the estimation of pig movement, this paper proposes a method that integrates optical flow with a multi-object tracking algorithm to assess pig movement, which is a robust and accurate approach tailored to quantify the intricate locomotion patterns of pigs. The adoption of optical flow as a cornerstone for characterizing pig movement is well founded given its ability to delineate pixel-level displacements between consecutive frames. Compared with bounding boxes, optical flow can avoid the aforementioned negative effects. At the same time, the multi-object tracking algorithm can distinguish the unique trajectories of each individual pig, allowing for extracting the movement of each pig from the optical flow. In response to the two key points mentioned before, we select a state-of-the-art multi-object tracking model, ByteTrack [15], as the tracking algorithm in our method. ByteTrack is a well-known multi-object tracking model that has demonstrated exceptional performance in scenarios characterized by severe occlusions. Severe occlusion is quite common in surveillance videos of pigs, and that is the reason why we adopt ByteTrack. As for optical flow, we explore several ways to compute it between two consecutive frames. We first employ the dense optical flow function [16] in the OpenCV library to obtain optical flow, while the performance is unsatisfactory due to the limitation of its primary assumption. We then turn to the deep learning method, considering the rapid progress in this research field. The two most representative models, FlowNet [17] and Skflow [18], are tested in our method. Our experimental endeavors conclusively demonstrate the superior performance of Skflow in optical flow estimation, substantiating its well-deserved status as the model of choice. It is worth noting that the optical flow estimation method and the tracking algorithm employed in this paper are not the only approaches. We select the appropriate methods based on our experimental results. A comprehensive exposition of the selection criteria underpinning optical flow computation is thoughtfully presented in Section 4.3.

The video dataset of pigs employed in our experimental inquiries is sourced from the dataset introduced in [19], owing to the ongoing development status of our data collection infrastructure. This dataset comprises a meticulously annotated collection of 1200 frames. However, in the quest to foster the training of a robust multi-object tracking model, this quantity is deemed insufficient. To overcome this constraint, we adopt a data augmentation strategy. Specifically, we subdivide an extensive video sequence into four discrete sub-sequences, proceeding to conduct annotations for approximately 1800 images within each of these delineated segments. This concerted effort led to the annotation of approximately 7200 images in total. It is pertinent to highlight that the annotation format adheres meticulously to the standardized conventions characteristic of multi-object tracking datasets, aligning seamlessly with the established format employed within the publicly accessible Multiple Object Tracking dataset [20].

The contributions of this paper are summarized as follows:

- This paper proposes to adopt optical flow as a cornerstone for estimating pig movement. Compared with the previous methods of only relying on tracking results, optical flow is not affected by fluctuations in the size of bounding boxes, leading to a more accurate and robust estimation of pig movement.
- This paper systematically dissects and elucidates the limitations inherent in the method of using bounding boxes and makes an extensive exploration of diverse methodologies employed in optical flow computation. This exhaustive analysis collectively serves as a foundational resource to propel the field of pig movement assessment toward greater understanding and precision.
- Considering that there are few pig tracking datasets available for related research, we make our annotated dataset open source, facilitating the progress of this research field.

The remainder of this paper is structured as follows. Section 1 introduces the background of livestock farming, problems in the estimation of pig movement, and our proposed method for assessing pig movement. Section 2 offers a brief review of applications of AI technologies in livestock farming and highlights the differences between the methodology presented in this paper and existing methods. Section 3 includes introductions of datasets we use for experiments, descriptions of the multi-object tracking algorithm, and the exploration of computing optical flow for estimating pig movement. In Section 4, we present experimental results of pig tracking and the calculation of pig movement. Moreover, a comprehensive discussion about several methods of computing optical flow is also included. Section 5 draws conclusions on the whole paper and clarifies the future directions of our work.

## 2. Related Works

In this section, we first offer brief reviews on the recent progress of precision livestock farming based on computer vision techniques, including detection, tracking, and behavior recognition for pig farming. Then, we highlight the existing methods of pig movement estimation and clarify the differences between the proposed method with these works.

### 2.1. Applications of Computer Vision Technologies in Pig Farming

The rapid advancement of computer vision technology has ushered the pig farming industry into an era of non-contact and automated breeding. Many classic computer vision tasks have been adapted and applied to the pig farming sector, providing a foundation for pig health monitoring and breeding management decisions. In terms of pig detection applications, Bo et al. [21] proposed a real-time pig detection system based on infrared cameras that effectively mitigates the issues of infrared reflection in pig farms. This system comprises a data collector for gathering infrared images, a preprocessor for converting noisy images into clean ones, and a detector for pig detection. The preprocessor employs U-Net and Generative Adversarial Networks (GAN) for feature extraction and is trained on paired clean datasets and datasets with simulated noise. Lei et al. [22] introduced a non-contact machine vision method where Mask R-CNN and UNet-Attention were implemented for sow target perception in complex scenarios. Ding et al. [23] proposed a method named FD-CNN to detect the regions of the active piglets based on YOLOv5s model. They employed a detection model to predict the area occupied by active piglets and then estimated the overall average activity level of piglets during the lactation period by calculating the ratio of this area to the total area occupied by all piglets. This analysis was used to study the variations in piglet activity.

Regarding pig tracking, Guo et al. [24] proposed a weighted association algorithm combined with two multi-object tracking models to improve the tracking performance. Liu et al. presented a new method to track individual trajectories for pigs where the tracking model was based on DeepLabCut and the trajectory optimal clustering was achieved by kernel principal component analysis. To measure the number and types of social encounters for pigs, Wutke et al. [25] developed a framework for the automated identification of social contacts in which Convolutional Neural Networks (CNN) and Kalman Filter were employed to recognize social contacts in the form of head–head and head–tail contacts. In terms of automated pig monitoring, Wang et al. [26] proposed a one-shot tracker to solve the re-identification problem in pig tracking. This method jointly trained detection models and re-identification models and combined re-identification features and IoU for matching.

To diagnose the productivity, health, and welfare of pigs, plenty of behavior recognition methods have been developed based on pig detection and tracking. Tu et al. [27] achieved pig behavior recognition based on tracking algorithms where the behavior identification of each pig was based on tracking results. Hao et al. [11] proposed a deep mutual learning enhanced two-stream method consisting of two mutual learning networks for identifying pig behaviors. In their approach, two mutual learning networks were able to extract rich appearance and motion features, improving performance. To recognize the aggressive behavior of group-housed pigs, Gao et al. [12] presented a hybrid model combining CNN and Gated Recurrent Unit (GRU) to extract behavior features, and a specific spatiotemporal attention mechanism was added into the model to better classify the behaviors. Ji et al. [28] utilized a temporal shift module inserted into four different CNN networks to automatically recognize pig behaviors. The whole model was efficient, without extra parameters and complexity. All these applications of computer vision techniques are beneficial to pig farming, improving the efficiency of management and reducing labor costs.

### 2.2. Pig Movement Estimation

The daily activity intensity of pigs is one of the crucial indicators for measuring their health. There are two kinds of mainstream methods to automatically assess pig movement. One method is to first identify pig behavior and then count the occurrences of each behavior [9,10,12]. These methods primarily concentrate on static behaviors and fail to provide quantified assessments of the current and cumulative movements of individual pigs, i.e., how far each pig moves. The other method harnesses tracking algorithms within the domain of computer vision to monitor the positions of individual pigs and subsequently assess pig movement using the center point of bounding boxes [8,13,14]. As we explained before, the size of bounding boxes is easily changed during the tracking process; thus, it is not reliable to be used for estimating the actual movement of each pig. On the contrary, the proposed method integrates optical flow with a tracking algorithm where the optical flow shows the movement of all pigs and the tracking results provide the bounding box information of each pig, beneficial to compute the movement of each pig. In this way, movement estimation and bounding boxes are decoupled, and movement calculation does not rely on the size of bounding boxes. Furthermore, the optical flow can reflect pig motions of different parts, which provides more fine-grained information for assessing the health status of pigs.

## 3. Materials and Methods

**Figure 2:**
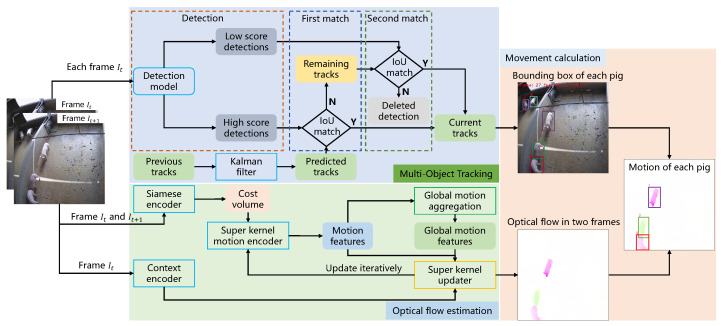
Schematic depiction of the proposed method’s operational pipeline for the estimation of pig movement. The proposed method mainly contains three modules, which are a tracking model, an optical flow estimation model, and a movement calculation module. The input data are video sequence frames. The tracking model takes each frame as input and outputs individual tracks for each pig. Meanwhile, the optical flow estimation model takes two consecutive frames as input and generates optical flow information between these two frames. Finally, the movement calculation module first maps the individual track of each pig on the optical flow to obtain every pig’s optical flow information and then calculates the movement of each pig.

This section presents a comprehensive overview of our methodology. The proposed approach can be succinctly distilled into three key steps, as depicted in Figure 2. First, a multi-object tracking model that takes two consecutive frames as input is employed to generate individual tracks for each pig in the video sequence. Here, we adopt the state-of-the-art tracking model, ByteTrack [15], as our tracking model. The main superiority of this model is that it contains two matching processes that match all bounding boxes as much as possible, whether they are high-confidence bounding boxes or low-confidence ones. Specifically, for frame $I_{t+1}$, the detection model takes the frame as input and generates bounding boxes for each pig. Meanwhile, the Kalman Filter [29] uses the previous tracks in frame $I_{t}$ to predict new tracks in frame $I_{t+1}$. Then, the bounding boxes with high scores and these new tracks participate in the first matching process, where the matched tracks are classified as current tracks and the unmatched tracks are listed as remaining tracks. The second matching process takes the remaining tracks and the bounding boxes with low scores as input, and then the matched tracks are seen as current tracks, while the unmatched bounding boxes are deleted directly. The detailed matching process is provided in Section 3.2.

Second, an optical flow estimation module is tasked with computing the optical flow between two successive frames, effectively capturing dynamic motion information. The estimation of optical flow is principally achieved through two distinct categories of methods: OpenCV-based techniques and deep learning-based approaches. Based on the experimental results, we finally choose the Skflow model [18] with the best performance as our optical flow estimation module. The siamese encoder in the Skflow model takes two consecutive frames $I_{t}$ and $I_{t+1}$ as input and outputs a cost volume. This cost volume is sent to a super kernel motion encoder to output motion features, and a global motion aggregation module generates global motion features. The motion features, the global motion features, and context features of frame $I_{t}$ from a context encoder are fed into a super kernel updater to produce optical flow. After certain iterations, the super kernel updater outputs a final refined optical flow.

Lastly, a pig movement calculation module leverages pig tracks generated by the tracking model and optical flow between two consecutive frames to obtain the individual optical flow of each pig, achieved by mapping bounding boxes to optical flow, and finally computes the movement of each pig. It is worth noticing that the tracking model and the optical flow estimation model adopted by the proposed method are not the only options, as we decided based on the experimental results. In the subsequent sections, we provide a detailed exposition of each facet comprising our proposed methodology.

### 3.1. Dataset Descriptions

**Figure 3:**
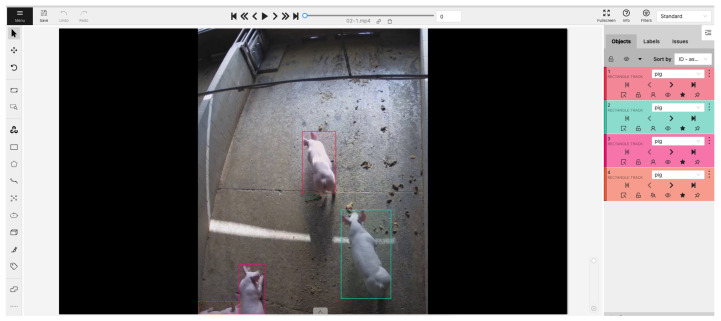
An example of using CVAT to annotate images. Different colorful boxes indicate different pigs. Dash box means there are occlusions.

The dataset employed in this paper is sourced from [19] due to the ongoing development status of data collection infrastructure. It is worth noting that the dataset provided in [19] primarily focuses on cross-camera tracking and comprises a relatively modest pool of training data, consisting of a mere 1200 annotated frames. To address the requirements of our research and expand our dataset, we adopt a practical approach. We select an extended video sequence, partitioning it into four distinct sub-sequences, each with a duration of 2 min. The original frame rate of each sub-sequence is 30 frames per second, which is subsequently reduced to 15 using FFMPEG software (https://ffmpeg.org/, accessed on 25 August 2023). Consequently, each sub-sequence yields a total of 2×60×15=1800 images. In total, our dataset encompasses 7200 images. For the crucial task of data annotation, we utilize the open-source labeling software known as CVAT (https://www.cvat.ai/, accessed on 25 August 2023). A representative example of the annotation process is depicted in Figure 3. Notably, the annotation format generated by CVAT adheres to the standardized conventions observed in multi-object tracking datasets [20]. The respective sub-sequences feature varying pig populations, with pig counts per sequence amounting to 4, 6, 8, and 7, respectively.

### 3.2. Pig Tracking Model

The tracking model is responsible for generating individual tracks for each pig across the video sequence, and the quality of tracking performance is crucial in determining whether the corresponding pig’s motion can be accurately extracted from the optical flow data. Therefore, our tracking model is based on state-of-the-art multi-object tracking, ByteTrack [15], and it is retrained on our pig dataset. ByteTrack is composed of two essential components: an object detection part and an IoU matching component, as illustrated in Figure 2. In alignment with established multi-object tracking paradigms, ByteTrack leverages an efficient detector that strikes an optimal balance between detection performance and computational speed. The ByteTrack model adopts the YOLOX series [30] for object detection, embracing an anchor-free approach and separating the detection head from the label assignment process. This design choice yields a significant enhancement in detection accuracy and processing speed. In contrast to earlier tracking algorithms such as SORT [31], DeepSORT [32], QDTrack [33], and FairMOT [34], ByteTrack exhibits superior performance in multi-object tracking tasks, particularly excelling in scenarios characterized by severe occlusion. One of ByteTrack’s distinctive strengths lies in its object association strategy. It not only pairs bounding boxes with high detection scores, but also effectively associates detections of occluded objects, whose detection scores often fall below a predefined threshold in the second matching phase. This approach sets ByteTrack apart from other tracking algorithms. In consideration of the prevalent usage of the YOLOX model for the detection component, a detailed emphasis is placed on the matching component. The matching process is succinctly summarized in Algorithm 1, adhering to the original paper’s formulation. The process begins by segregating detections into high-score detections ($D_{high}$) and low-score detections ($D_{low}$) (Lines 5 to 10). Subsequently, it employs the Kalman Filter [29] to predict the new positions of each track in T within the current frame (Lines 11 to 14). The first association is accomplished by matching high-score detections ($D_{high}$) with tracks in T (Lines 15 to 17). Unmatched detections and tracks are then allocated to $D_{remain}$ and $T_{remain}$, respectively. A second association is performed between low-score detections ($D_{low}$) and unmatched tracks in $T_{remain}$ (Lines 18 to 19). Post these two association steps, any remaining unmatched detections are considered background and consequently removed. As for the unmatched tracks ($T_{re-remain}$) after the second association, they are retained for a predefined number of frames, typically 30, before being discarded. Finally, unmatched high-score detections in $D_{remain}$ are initialized as new tracks (Lines 21 to 22). This two-tiered association process not only optimizes track-to-detection matching, but also significantly enhances overall tracking performance.
**Algorithm 1** Matching process in ByteTrack [15]**Input:** A detection of the sequence D, detection score threshold τ**Output:** Tracks T of the video  1:Initialization: T←⌀  2:**for** $D_{k}$ *in* D **do**  3:   $D_{high}\leftarrow ⌀$  4:   $D_{low}\leftarrow ⌀$  5:   **for** *d in* $D_{k}$ **do**  6:     **if** *d.score* > τ **then**  7:        $D_{high}\leftarrow D_{high}\cup d$  8:     **else**  9:        $D_{low}\leftarrow D_{low}\cup d$10:     **end if**11:   **end for**12:   **for** *t in* T **do**13:     *t* ← *KalmanFilter*(*t*)14:   **end for**15:   Associate T and $D_{high}$ using IoU matching16:   $D_{remain}\leftarrow$ remaining object boxes from $D_{high}$17:   $T_{remain}\leftarrow$ remaining tracks from T18:   Associate $T_{remain}$ and $D_{low}$ using IoU matching19:   $T_{re-remain}\leftarrow$ remaining tracks from $T_{remain}$20:   T←T \ $T_{re-remain}$21:   **for** *d in* $D_{remain}$ **do**22:     T←T∪d23:   **end for**24:**end for**

### 3.3. Estimation of Pig Movement

In this subsection, we provide a comprehensive exposition of our approach to estimating pig movement. We begin by introducing the conventional methods that rely on bounding boxes, indicating its drawbacks. After that, we provide a detailed explanation of our proposed optical flow-based approach.

It is noteworthy that the conventional literature [13] predominantly relies on calculating pig movement, denoted as *M*, by measuring the distance between the center points of two consecutive frames, as defined in Equation (1).

(1) $$M=\sqrt{{\left(x_{c}^{t}-x_{c}^{t+1}\right)}^{2}+{\left(y_{c}^{t}-y_{c}^{t+1}\right)}^{2}},$$

where $\left(x_{c}^{t},y_{c}^{t}\right)$ and $\left(x_{c}^{t+1},y_{c}^{t+1}\right)$ are the coordinates of the center point within the bounding boxes in frame *t* and frame t+1, respectively. As previously emphasized, this method is notably contingent on the size of predicted bounding boxes. However, the tracking model does not guarantee fixed bounding box sizes when pigs are stationary. Furthermore, variations in bounding box size occur when only the pig’s head is in motion, as opposed to their feet, and when pigs are occluded, leading to a significant reduction in bounding box dimensions. These inherent limitations associated with center point-based calculations engender inaccuracies in recording pig movement, thereby adversely affecting the management and decision-making processes within pig farming.

**Figure 4:**
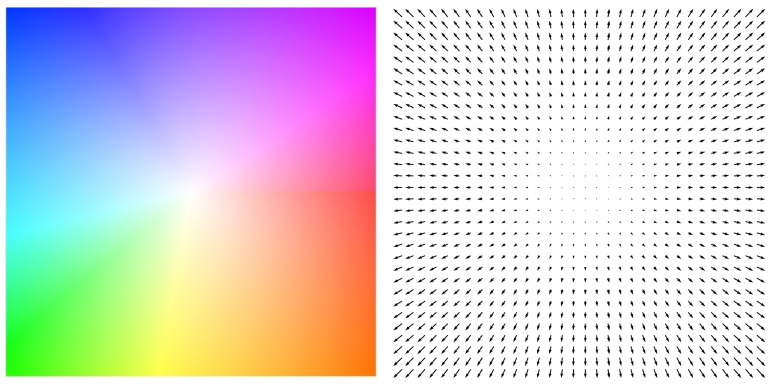
An example of optical flow spreading out from the center.

Therefore, this paper advocates the utilization of optical flow information as a means to estimate pig movement. Optical flow essentially provides a visual representation of the apparent motion exhibited by objects between successive frames, resulting from their spatial displacement. A visualization of optical flow is shown in Figure 4. The image on the right represents sparse optical flow visualization, where the direction of the arrows indicates the direction of pixel movement, and the length of the arrows represents the magnitude of pixel displacement. The image on the left represents the visualization of dense optical flow, where colors represent the direction of pixel movement, and the intensity of colors indicates the magnitude of pixel displacement.

To elucidate, considering I(x,y,t) as the intensity of image I at time *t*, and allowing Δt representation of the time interval, the movement of pixel (x,y), denoted as (Δx,Δy), can be determined by following the *brightness constancy constraint* hypothesis, as formulated in Equation (2).

(2) I(x,y,t)=I(x+Δx,y+Δy,t+Δt),

where (Δx,Δy) is the optical flow at pixel (x,y). The predicted optical flow is a vector that encompasses both the magnitude of pixel displacement and the direction of motion. Given that optical flow pertains to pixel-level information, effectively conveying the movement of each pixel, there are two ways to compute pig movement. The first way is to designate a specific point as the pig’s representative and then take the distance moved by this point as the movement of a pig. One feasible approach is to select the center point denoted as $\left(x_{c}^{0},y_{c}^{0}\right)$ from the initial track to represent the pig. Consequently, when the pig exhibits motion across frames, the corresponding movement *M* can be calculated using Equation (3). This approach ensures accurate pig movement calculations, even when a pig’s body transitions from partial occlusion to complete visibility.

(3) $$M=\sqrt{{\left(\Delta x\right)}^{2}+{\left(\Delta y\right)}^{2}},$$

where $\left(\Delta x,\Delta y\right)=F\left(x_{c},y_{c}\right)$, and F indicates the predicted optical flow. Suppose that (Δx,Δy) is the optical flow of the representative point $\left(x_{c}^{t},y_{c}^{t}\right)$ at frame *t*. The coordinate of the representative point in the next frame is computed by Equation (4).

(4) $$\left(x_{c}^{t+1},y_{c}^{t+1}\right)=\left(x_{c}^{t}+\Delta x,y_{c}^{t}+\Delta y\right).$$

This method only focuses on one point and ignores the movement of other parts of a pig. For instance, if a pig just shakes its head, then this movement is ignored. On the contrary, the second way is to consider the movement of the whole pig. In the case that movement of other parts, such as the head and ears whose moving directions are consistent, are being considered, the movement is the average of optical flow inside the bounding box, as shown in Equation (5).

(5) $$M=\frac{1}{h\times w}\sum_{(u,v)\in B}{\left||F\left(u,v\right)|\right|}_{2},$$

where B is a set of pixel positions inside a bounding box. *h*, *w* indicate the height and width of the bounding box. In our paper, we take the second way to compute the movement of all parts of a pig.

Based on the aforementioned analysis, the crucial factor in obtaining an accurate estimation of pig movement lies in the methodology of obtaining optical flow. We explored various approaches, primarily classified into two categories: a dense optical flow function from the OpenCV library, and those rooted in deep learning techniques. The OpenCV library provides a dense optical flow function [16], which produces a dense optical flow, encompassing optical flow data for all pixels. The output consists of a vector with two channels, representing optical flow along the x-axis and the y-axis, respectively. Each channel includes magnitude and direction information. Although this function is convenient to implement, it is susceptible to environmental factors. Since our input images are derived from real-world settings, the function may not adhere to the *brightness constancy constraint* hypothesis, causing exhibit suboptimal performance. Considering the limitations of the OpenCV-based approach, we turn to deep learning models for optical flow estimation. These models have been extensively trained on publicly available datasets and can be directly applied to infer optical flow within our pig dataset. We select two prominent deep learning models, FlowNet [17] and Skflow [18], for our experiments. FlowNet, as the pioneer in using CNN for optical flow estimation, formulates optical flow estimation as a supervised task and employs two distinct architectures. It has been trained on synthetic datasets and demonstrates impressive performance on realistic datasets. On the other hand, Skflow utilizes a CNN architecture to mitigate the impact of occlusions and leverages super kernels to enhance its performance. As of the current state of the art, it stands as the best model in optical flow estimation. According to our experimental results, Skflow is the best in terms of performance, followed by the OpenCV function, while FlowNet has the poorest performance. Detailed experimental comparisons are presented in Section 4.3. Therefore, this paper chooses Skflow as our optical flow estimation model, while it is worth noting that Skflow is not the only option available.

## 4. Experiments and Discussions

This section commences with an overview of our experimental setup, encompassing common hyper-parameters for deep learning models, dataset partitioning, and the metrics used for estimation. Subsequently, we present the results of pig tracking performance and the outcomes of pig movement calculations using various optical flow methods. Lastly, we engage in a comprehensive discussion concerning the methodologies employed for estimating pig movement.

### 4.1. Implementation Details

In our experiments, we employ four annotated video sequences, with the initial three sequences allocated for training and validation, while the final sequence serves as the test dataset. We follow the data splitting convention established by multi-object tracking dataset [20], where the training data are evenly divided into training and validation subsets to facilitate parameter tuning. All frames are resized to a uniform size of 640×640. For the detection model, we set the detection threshold at 0.1 and the non-maximum suppression threshold at 0.5. During the matching process, we utilize a matching threshold of 0.5 and cap the maximum number of retained tracks at 100. Regarding the two deep-learning optical flow models, only the inference process is conducted, and all parameters remain consistent with those defined in their original papers. We employ the SGD optimizer with a weight decay of $5\times 10^{-4}$ and a momentum of 0.9. The initial learning rate is set to 0.001, employing a one-epoch warm-up and a cosine annealing schedule. The overall training spans 100 epochs, with the remaining parameters in accordance with ByteTrack’s training settings [15].

The evaluation of tracking performance encompasses Higher-Order Tracking Accuracy (HOTA) [35], CLEAR metrics [36], and Identity metrics [37]. HOTA comprehensively considers the accuracy of both object detection and object tracking, balancing the performance of precise detection, association, and localization into a unified metric for tracker comparison. CLEAR metrics focus on the detection performance of tracking models, including True Positives (CTP), False Positives (CFP), False Negatives (CFN), and Multiple Object Tracking Accuracy (MOTA). CTP is the number of correctly detected samples. CFP is the number of incorrectly detected samples, i.e., non-existent samples incorrectly detected. CFN is the number of true samples that are not detected, i.e., the number of missed detection samples. Identity metrics concentrate on evaluating the performance of tracking algorithms in maintaining sample identity consistency, including Identity True Positives (IDTP), Identity False Positives (IDFP), Identity False Negatives (IDFN), Identity Switches (IDSW), and Identity F1 score (IDF1). IDTP indicates the number of samples that are correctly detected and whose identity is also correctly associated. IDFP refers to the number of samples that are incorrectly marked as a specific sample. This occurs when the algorithm identifies a non-existent sample or a false sample as a known sample. IDFN is the number of samples correctly detected but not properly associated with their known identities. IDSW is the number of sample identity switches during tracking. IDF1 is a metric formulated by Equation (6) where it balances precision and recall for identity preservation and places a higher emphasis on association performance.

(6) $$IDF1=\frac{2\times IDTP}{2\times IDTP+IDFP+IDFN}.$$

In contrast, MOTA, which is formulated by Equation (7), gauges the accuracy of multi-object tracking by considering CFP, CFN, and IDSW, highlighting detection performance.

(7) $$MOTA=1-\frac{\sum_{t}\left(CFN_{t}+CFP_{t}+IDSW_{t}\right)}{\sum_{t}GT_{t}},$$

where GT is the number of true samples and *t* is the time frame.

### 4.2. Performance of Pig Tracking

The performance of the tracking algorithms on our pig dataset is presented in Table 1. We trained four commonly used multi-object tracking models, Sort [31], DeepSort [32], Tracktor [38], and ByteTrack [15], on our pig dataset. Sort, DeepSort, and Tracktor share the same detector, Faster R-CNN [39], while differing in association methods. Sort achieves association by bounding box overlapping based on the Kalman Filter and the Hungarian algorithm. DeepSort is an extension of Sort, adding a re-identity (ReID) model that usually is a deep learning model, to associate samples. On the contrary, Tracktor avoids complex data association problems in many cases by directly using the output of the detector. Additional data association steps are only required when a sample is lost or a new sample appears. The results in Table 1 show that ByteTrack and DeepSort generally outperform the other two tracking models. In particular, ByteTrack has a MOTA of 98.8%, surpassing DeepSort by 3%, showing its superiority in tracking accuracy. ByteTrack has a CTP of 7930, a CFN of 5, and a CFP of 87, which means that during the tracking process, ByteTrack correctly detected 7930 true samples, only took 87 non-existent samples as true samples, and missed 5 true samples. Compared with DeepSort which has a CTP of 7836, a CFN of 99, and a CFP of 229, ByteTrack has much better tracking accuracy. However, ByteTrack has an IDF1 of 95.0%, 2.2% lower than DeepSort, showing that DeepSort has a stronger ability for identity preservation. ByteTrack has an IDTP of 7579, an IDFN of 356, and an IDFP of 438, indicating that during the tracking process, ByteTrack correctly detected and correctly associated 7579 samples, correctly detected 356 samples yet assigned wrong identities to them, and correctly detected 438 samples but incorrectly associated them with known identities. DeepSort instead has a higher IDTP count at 7799, surpassing ByteTrack, and it also has lower IDFN and IDFP counts, at 136 and 321, respectively, which are significantly lesser than those of ByteTrack. On the other hand, ByteTrack has an extremely smaller IDSW at 2 compared with DeepSort’s IDSW at 13. In terms of HOTA, ByteTrack is only 1.1% lower than DeepSort, showing a slightly inferior comprehensive tracking performance.

All these analyses show that compared with DeeoSort, ByteTrack performs better in terms of tracking accuracy, while it is slightly inferior in identity preservation ability. This fact is not consistent with the original ByteTrack paper. We argue the reason is that our testing scenarios have few occlusions due to a small number of pigs. As a result, the association method using ReID performs better than the association method of IOU metrics. When it comes to scenarios that have severe occlusions, the ReID model struggles to extract discriminate appearance features for a target, resulting in poor association performance, which can be drawn from the ByteTrack paper. In our case, we choose ByteTrack as the tracking model for the proposed method. We cannot make sure there are no occlusions in the pig farm; more importantly, we pay more attention to the tracking accuracy of the tracking algorithm to ensure that every pig can be correctly detected. As for shortcomings of identity preservation ability, it is possible to use other methods to overcome them. For instance, it is feasible to add an extra pig face recognition model to assign a unique and permanent ID to each pig.

**Figure 5:**
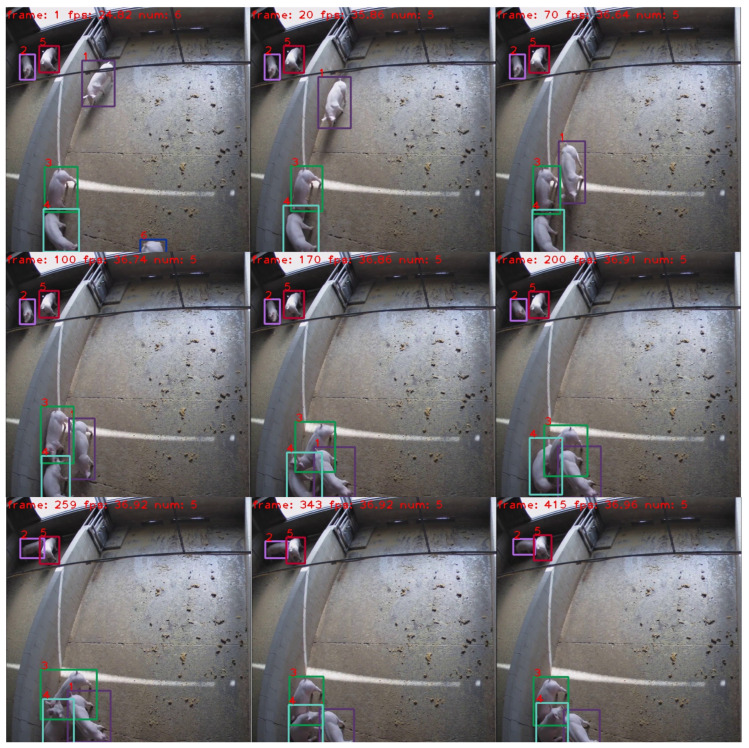
Visualizations of the ByteTrack model on our pig dataset.

In addition, we also conduct experiments to compare the tracking performance of different detection models of ByteTrack, as illustrated in Table 2. ByteTrack employs YOLOX series models [30] for object detection. There are four different types of models in the YOLOX series, which are YOLOX-X (Extra Large), YOLOX-L (Large), YOLOX-M (Medium), and YOLOX-S (Small). YOLOX-S is the smallest model designed to achieve higher speeds while making some compromises on performance. It is suitable for resource-constrained devices or application scenarios that require fast processing. The YOLOX-M model provides a middle option between parameter size and detection speed. It is more accurate than YOLOX-S, but slightly slower for scenes that require a balance of speed and performance. The YOLOX-L model has a larger model and offers better performance, while it is slower than YOLOX-S and YOLOX-M in terms of processing speed. It is suitable for applications that require a higher level of performance. YOLOX-X is a model with the biggest parameter size, the best detection performance, and the slowest detection speed. It is designed for the case that requires extremely high detection performance. The results in Table 2 indicate that YOLO-X has the best performance both in tracking accuracy and identity preservation, in which HOTA, MOTA, IDF1, and IDSW display the values of 83.1%, 98.8%, 95.0%, 2, respectively. Although YOLOX-X has the largest parameter size of 99 M, the Frame Per Second (FPS) value of YOLOX-X is about 38, bigger than 30, which means that the tracking model can be run in real time. Considering its excellent performance and real-time tracking speed, we take YOLOX-X as the detection model of ByteTrack. To further illustrate the tracking quality, visualizations of the tracking results are provided in Figure 5, demonstrating effective tracking of individual pigs.

### 4.3. Calculation of Pig Movement

**Figure 6:**
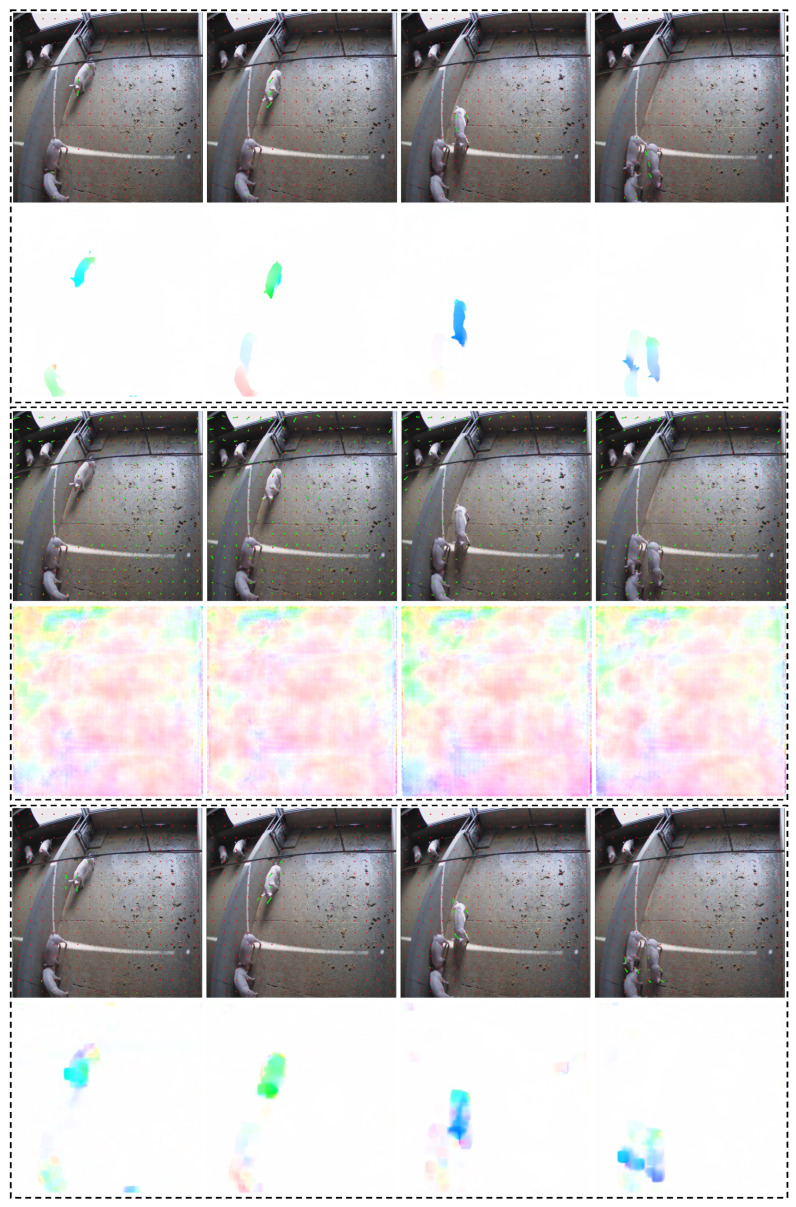
Visualizations of predicted optical flow. From top to bottom, the three blocks are the results of Skflow, FlowNet, and the OpenCV function, respectively. In each block, images in the first row are sparse optical visualizations, and images in the second row are dense optical flow.

As detailed in Section 3.3, the crucial aspect of employing optical flow for the estimation of pig movement lies in the acquisition of precise optical flow information. In this subsection, we visualize some predicted optical flow images and compare their qualities. The visualization images are presented in Figure 6. In Figure 6, from top to bottom, there are three blocks indicating the results of Skflow [18], FlowNet [17], and the OpenCV function [16], respectively. In each block, images in the first row are sparse optical visualizations where the sampled pixels are denoted as red, the green arrows represent the moving direction, and the length of the green arrows indicates the displacement. The images in the second row are dense optical flow visualizations, where the color indicates the moving direction and the intensity of color denotes the displacement, referring to Figure 4.

From the visualizations, we can see that Skflow has the best performance in estimating optical flow, followed by the OpenCV function, and FlowNet has the worst performance. In the sparse visualizations, FlowNet shows optical flow in the floor area, represented by green arrows. On the contrary, the OpenCV function and Skflow have correct predictions in that area. In terms of dense optical flow images, the predictions from FlowNet are full of noise, resulting in serious optical flow errors. On the contrary, the OpenCV function and Skflow can generate relatively clean backgrounds, only showing the motion of pigs. Comparing the OpenCV function and Skflow, the optical flow produced by Skflow can retain the shape of objects, while the results of the OpenCV function show blurred shapes, indicating the superiority of Skflow on the edge position. The dense optical flow visualizations of Skflow show different colors and intensities for each pig, which indicates its capability to capture the motions of pigs, even the multifarious motions in different parts. This attribute enables the provision of finer-grained movement information. The visualization images underscore the effectiveness of employing an accurate optical flow estimation model for generating high-quality optical flow representations of pig movement.

**Figure 7:**
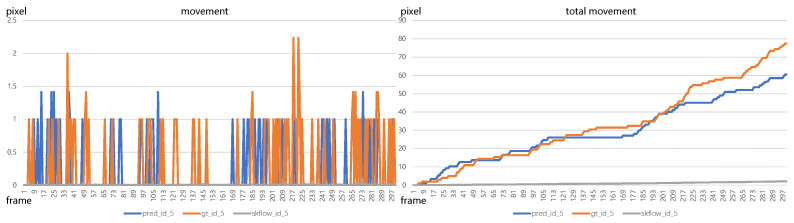
The comparisons of using bounding boxes to calculate pig movement where bounding boxes are obtained from tracking results and annotation, respectively.

Based on optical flow data, it is convenient to calculate the pig movement between two consecutive frames. We first present the movement calculated by bounding boxes from tracking results and annotation, as shown in Figure 7. The left image in Figure 7 shows the movement between each frame and the right image indicates the accumulative movement. It is evident that there is a substantial disparity between using bounding boxes obtained from tracking results and using those from annotations. The bounding boxes in the annotation are strictly restrained to just cover the edge of the pigs. However, the tracking algorithm cannot ensure the predicted bounding boxes with a fixed size, i.e., the same size of the bounding box as in annotation, causing errors in calculating pig movement. All of these drawbacks can be avoided by employing optical flow. The curve of total movement calculated by the optical flow estimation model, Skflow, shows a very small accumulative movement of the pig (id_5), compared with bounding boxes. From the tracking visualizations in Figure 5, we can see that the pig (id_5) is actually keeping static, which is consistent with the estimation result using optical flow. Consequently, the incorporation of optical flow models to calculate pig movement emerges as a favorable choice, as it eliminates dependence on bounding boxes and mitigates the adverse effects arising from bounding box size variations.

**Figure 8:**
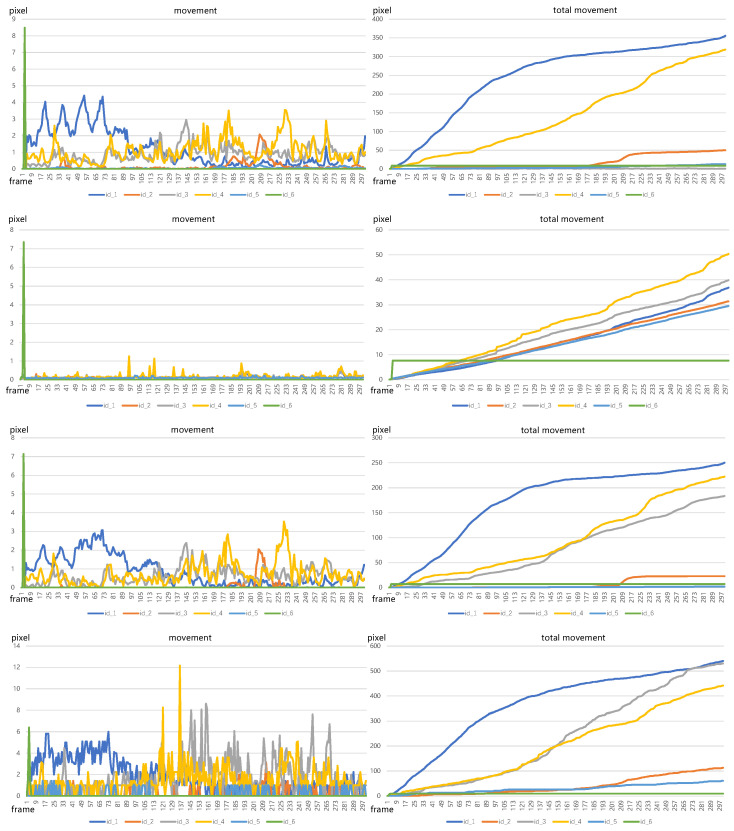
Pig movement is calculated by different methods. From top to bottom, the figures of pig movement are calculated by the OpenCV function, FlowNet, Skflow, and bounding boxes, respectively. The images on the left side are the movement between two consecutive frames and the right ones are accumulative movement.

Figure 8 displays the pig movement results calculated by optical flow and bounding boxes, all measured in pixels. From top to bottom, the figures are the results calculated by the OpenCV function, FlowNet, Skflow, and bounding boxes, respectively. The left-side images are the movement of each pig between frames, and the right ones are the accumulative movement of each pig. In terms of results based on optical flow data, the movement from the OpenCV function and Skflow are similar since they have semblable optical flow predictions, while the results from FlowNet have huge differences from those of the OpenCV function and Skflow due to the inaccurate optical flow predictions. As for total movement, the OpenCV function and Skflow have the same movement trends for each pig, only differing in values. The reason is that although the OpenCV function has the ability to capture the movement of each pig, the performance on the edge place is not as good as that of Skflow, resulting in large error motion estimation, such as the optical flow estimation of the pig with id_3. This pig overlaps with other pigs, leading to incorrect movement estimation. Comparing the movement estimated by optical flow and bounding boxes, we can find that the method of using bounding boxes has large errors in the static pigs, such as pigs with id_2, id_3, and id_5.

The tracking visualizations in Figure 5 show that the pigs with id_2 and id_5 are almost keeping static, and their movement should be very small. The total movement curves of those two pigs from Skflow indicate small values, while the corresponding curves from bounding boxes present large movement values. On the other hand, due to occlusion by other pigs, the detection box size for the pig with id_3 varies significantly, resulting in a sharp increase in total motion magnitude. Therefore, the total movement value of this pig computed by bounding boxes is much larger than that of its counterpart from Skflow. These errors are caused by the variations in bounding box size, which reveals the shortness of employing bounding boxes to estimate pig movement. On the contrary, the curves from Skflow are consistent with the observations, verifying the effectiveness and accuracy of adopting optical flow to estimate pig movement.

### 4.4. Discussions

**Analysis on employing optical flow.** While our proposed method, which combines a multi-object tracking algorithm with optical flow for pig movement estimation, outperforms methods based on center points of bounding boxes, also known as only using tracking algorithms, it is not without its drawbacks. Pigs exist in a three-dimensional (3D) space, and their movement should ideally be calculated in a 3D rather than a two-dimensional (2D) space. Twp-dimensional optical flow methods are limited in that they neglect motion directions perpendicular to the camera plane. This limitation arises from the inherent loss of information when transitioning from 3D to 2D. Tracking pigs in a 3D space requires more complex networks and matching mechanisms, leading to higher computational resource demands. While it is conceivable that 3D tracking algorithms could yield more accurate pig movement estimations, the associated deployment costs are considerably higher. Considering practicality and resource constraints, 2D tracking with optical flow estimation remains a more suitable approach. We contend that the margin of error between the movement estimated by optical flow and the actual motion is acceptable.

**Limitations of the proposed method and possible solutions.** As presented in Section 4.2, the tracking model of the proposed method, ByteTrack, is unable to achieve the best performance where HOTA, MOTA, and IDF1 should be as close to 100% as possible, and IDSW should be zero for estimating pig movement. In terms of tracking accuracy, i.e., the MOTA metric, it would be helpful to collect more data for training. Limited by annotation labor, the current training dataset only contains about 3600 images, much smaller than the public MOT dataset or the COCO dataset. We believe that the use of more training data is an effective measure to increase tracking accuracy. On the other hand, ByteTrack has a restricted ability for identity preservation. This disadvantage can be improved by adding an extra pig face recognition model. More specifically, the pig face recognition model can assign a unique and permanent ID to each pig. This permanent ID can be used as a piece of auxiliary feature information in the matching process. In this way, the numbers of IDFP and IDFN can be extremely reduced. Furthermore, even if the system restarts, the tracking ID can be consistent with the previous running.

## 5. Conclusions

In this paper, we provide both qualitative and quantitative analyses of the shortcomings of solely relying on tracking algorithms, i.e., based on bounding boxes, for pig motion calculation. To overcome the shortcomings, we propose a novel approach that combines optical flow with a multi-object tracking method to assess pig movement accurately. The optical flow can effectively capture the motion of objects and the multi-object tracking method can identify each pig instance. By combining optical flow and the tracking algorithm, it is easy to accurately estimate the movement of each pig. To select a tracking algorithm, we retrain four commonly used multi-object tracking models, Sort, DeepSort, Tracktor, and ByteTrack, on our pig dataset. The experimental results show that ByteTrack performs best in tracking accuracy while DeepSort has the best performance in identity preservation ability. Although ByteTrack has a slightly inferior identity preservation ability, we still choose it as the tracking algorithm for the proposed method, because it is more important to accurately track each pig for pig movement estimation and the drawbacks in identity preservation can be solved by adding an extra pig face recognition model. In our pursuit of precise optical flow, we conduct comprehensive experiments using an OpenCV function and two prominent deep learning models, FlowNet and Skflow. The results unequivocally highlight Skflow as the superior model for optical flow estimation, making it the optical flow estimation model in our approach. The visualized optical flow images produced by Skflow aptly convey the nuances of optical flow in our pig dataset and the predictions accurately capture instances where only specific parts of a pig, such as its head, are in motion. The movement curves show the superiority of adopting optical flow to using bounding boxes, emphasizing the effectiveness of optical flow in calculating pig motion. Our method effectively overcomes the limitations of existing techniques, which struggle to quantify pig movement and are susceptible to inaccuracies stemming from fluctuations in bounding box sizes. By incorporating optical flow, we not only obtain precise pig movement measurements between consecutive frames, but also accumulate this movement over defined time intervals. These capabilities furnish us with granular information that can significantly enhance decision-making and management processes in the context of pig farming. In our future work, on the one hand, we will concentrate on combining the pig face recognition model with ByteTrack to obtain the best tracking performance, which is also beneficial for accurate estimation of pig movement. On the other hand, we will attempt to explore a simple and efficient method for estimating pig movement in a 3D space, eliminating the movement error brought by transferring a 3D space to a 2D image space.

## Figures and Tables

**Table 1:** Results of four different tracking models on our pig dataset. ↑ means larger is better and ↓ indicates smaller is better. Bold values indicate the best evaluation.

Model	HOTA↑	MOTA↑	CTP↑	CFN↓	CFP↓	IDSW↓	IDF1↑	IDTP↑	IDFN↓	IDFP↓
Sort [31]	64.5%	95.8%	7866	69	254	10	61.5%	4939	2996	3181
DeepSort [32]	**84.2%**	95.8%	7866	13	254	13	**97.2%**	**7799**	**136**	**321**
Tracktor [38]	79.8%	95.8%	7836	99	229	3	90.7%	7255	680	810
ByteTrack [15]	83.1%	**98.8%**	**7930**	**5**	**87**	**2**	95.0%	7579	356	438

**Table 2:** Results of different detection models of ByteTrack. ↑ means larger is better and ↓ indicates smaller is better. Bold values indicate the best evaluation.

Model	HOTA↑	MOTA↑	IDF1↑	FP↓	FN↓	IDSW↓	Param.	FPS
YOLOX-X	**83.1**	**98.8**	**95.0**	87	**5**	**2**	99 M	37.5
YOLOX-L	77.8	87.2	93.2	17	996	**2**	54.15 M	42.83
YOLOX-M	77.7	87.6	93.4	**3**	977	4	25.28 M	51.64
YOLOX-S	72.8	83.5	87.2	244	1057	6	8.94 M	62.62

## Data Availability

The dataset in this paper is available upon request.

## Abbreviations

The following abbreviations are used in this manuscript:

AI	Artificial Intelligence
PLF	Precision Livestock Farming
IoT	Internet of Things
CNN	Convolutional Neural Networks
GAN	Generative Adversarial Networks
GRU	Gated Recurrent Unit
IoU	Intersection over Union
HOTA	Higher-Order Tracking Accuracy
MOTA	Multiple Object Tracking Accuracy
CTP	True Positives in CLEAR metrics
CFP	False Positives in CLEAR metrics
CFN	False Negatives in CLEAR metrics
IDTP	True Positives in Identity metrics
IDFP	False Positives in Identity metrics
IDFN	False Negatives in Identity metrics
IDSW	Identity Switches in Identity metrics
IDF1	Identity F1 Score in Identity metrics
ReID	Re-identity
3D	Three-dimensional
FPS	Frame Per Second
2D	Two-dimensional
